# The role of molecular subtypes and immune infiltration characteristics based on disulfidptosis-associated genes in lung adenocarcinoma

**DOI:** 10.18632/aging.204782

**Published:** 2023-06-13

**Authors:** Cui Qi, Jianmin Ma, Jinjin Sun, Xiaolin Wu, Jian Ding

**Affiliations:** 1 Department of Respiratory Medicine, Qingdao Women’s and Children’s Hospital, Qingdao, China; 2 Department of Cardiac Ultrasound, The Affiliated Hospital of Qingdao University, Qingdao, China; 3 Department of Operating Room, The Affiliated Hospital of Qingdao University, Qingdao, China; 4 Department of Orthopedics, The Affiliated Hospital of Qingdao University, Qingdao, China; 5 Cancer Institute, The Affiliated Hospital of Qingdao University, Qingdao University, Qingdao Cancer Institute, Qingdao, China; 6 Department of Neurology, The Affiliated Hospital of Qingdao University, Qingdao, China

**Keywords:** lung adenocarcinoma, prognostic model, disulfidptosis, immune infiltration, proliferation

## Abstract

Lung adenocarcinoma (LUAD) is the most common type of lung cancer which accounts for about 40% of all lung cancers. Early detection, risk stratification and treatment are important for improving outcomes for LUAD. Recent studies have found that abnormal accumulation of cystine and other disulfide occurs in the cell under glucose starvation, which induces disulfide stress and increases the content of disulfide bond in actin cytoskeleton, resulting in cell death, which is defined as disulfidptosis. Because the study of disulfidptosis is in its infancy, its role in disease progression is still unclear. In this study, we detected the expression and mutation of disulfidptosis genes in LUAD using a public database. Clustering analysis based on disulfidptosis gene was performed and differential genes of disulfidptosis subtype were analyzed. 7 differential genes of disulfidptosis subtype were used to construct a prognostic risk model, and the causes of prognostic differences were investigated by immune-infiltration analysis, immune checkpoint analysis, and drug sensitivity analysis. qPCR was used to verify the expression of 7 key genes in lung cancer cell line (A549) and normal bronchial epithelial cell line (BEAS-2B). Since G6PD had the highest risk factor of lung cancer, we further verified the protein expression of G6PD in lung cancer cells by western blot, and confirmed through colony formation experiment that interference with G6PD was able to significantly inhibit the proliferation ability of lung cancer cells. Our results provide evidence for the role of disulfidptosis in LUAD and provide new ideas for individualized precision therapy of LUAD.

## INTRODUCTION

Lung adenocarcinoma (LUAD) is a type of lung cancer that begins in the cells lining the lungs and typically develops in the outer regions. It is the most common type of lung cancer which accounts for about 40% of all lung cancers [1]. Risk factors for LUAD include smoking, exposure to secondhand smoke, exposure to certain chemicals or substances, a family history of lung cancer, and certain genetic mutations [2]. Treatment options for LUAD depend on the stage of the cancer, the patient’s overall health, and other factors. Treatment may include surgery, radiation therapy, chemotherapy, targeted therapy, or a combination of these approaches [3]. Concerning the fact that there may be no symptoms in the early stages in many cases, early detection and treatment are important to improve outcomes for LUAD [4].

Recent studies have found that in highly SLC7A11 expressed cells, abnormal accumulation of cystine and other disulfide occurs in the cell under glucose starvation, which induces disulfide stress and increases the content of disulfide bond in actin cytoskeleton, resulting in actin filament contraction, destruction of cytoskeleton structure, and cell death [5]. Unlike apoptosis and ferroptosis, this novel mode of cell death defined as disulfidptosis is not mitigated by other inhibitors of cell death, nor does it result from intracellular ATP depletion, but is enhanced by thiol oxidation reagents such as diamide [5]. Metabolic treatment of glucose transporter (GLUT) inhibitors can trigger disulfidptosis and inhibit the growth of cancers such as renal cell carcinoma. By inhibiting glucose uptake of cancer cells, GLUT inhibitors lead to decreased NADPH production and increased NADP+/NADPH ratio, which in turn leads to abnormal formation of actin-cytoskeletal protein disulfide bond and collapse of F-actin network, ultimately promoting the occurrence of disulfidptosis [5]. Additionally, the tumor microenvironment plays a major role in tumor formation and immune escape in lung cancer and influences the therapeutic responsiveness of LUAD [6]. Disulfidptosis has also been shown to have the potential to affect immune infiltration [7]. Because the study of disulfidptosis is in its infancy, its role in disease progression is still unclear.

Although the number of disulfidptosis associated genes (DAGs) explored is small, evidence of a potential relationship between DAGs and LUAD development exists. As a cystine/glutamate antiporter with cystine uptake specificity, SLC7A11 is a key acting molecule of disulfidptosis process. We note that high SLC7A11 expression in LUAD has been reported to be significantly increased and predicted an advanced clinical progression [8]. In KRAS-mutant LUAD, suppression of the SLC7A11/glutathione axis causes synthetic lethality [9]. In addition, the regulation of m6A methylation has been reported to be closely related to the action of SLC7A11 in LUAD. METTL3 promoted LUAD tumor growth by stabilizing SLC7A11 m6A modification [10]. Inhibition of m6A reader YTHDC2 promoted SLC7A11 expression and cystine uptake and its downstream antioxidant program, thus promoting LUAD tumorigenesis [11]. The above evidence suggests that SLC7A11 plays an important role in the development of LUAD tumors. In addition, significant correlations between SLC7A11 and a variety of cancer-infiltrating immune cells were reported, showing potential causality of SLC7A11 in regulation of T cell function in LUAD [8]. However, the role of disulfidptosis in LUAD involving SLC7A11 has not been reported.

In this study, we detected the expression and mutation of DAGs in LUAD using a public database. Cluster typing based on disulfidptosis gene was performed and differential genes of disulfidptosis subtype were analyzed. Subsequently, 7 differential genes of disulfidptosis subtype were used to construct a prognostic risk model, and the causes of prognostic differences were investigated by immune-infiltration analysis, immune checkpoint analysis, and drug sensitivity analysis. qPCR was used to verify the expression of 7 key genes in lung cancer cell line (A549) and normal bronchial epithelial cell line (BEAS-2B). Since G6PD had the highest risk factor of LUAD, we further verified the protein expression of G6PD in LUAD cells by western blot, and confirmed through colony formation experiment that interference with G6PD can significantly inhibit the proliferation ability of lung cancer cells. Our results provide evidence for the role of disulfidptosis in LUAD and provide new ideas for individualized precision therapy of LUAD.

## MATERIALS AND METHODS

### Acquisition and preprocessing of the LUAD dataset

The gene microarray and clinical information of LUAD were acquired from the publicly available Gene Expression Omnibus (GEO) and The Cancer Genome Atlas (TCGA) datasets. Following exclusion of LUAD samples with incomplete information regarding survival time, a total of 397 LUAD samples and 567 samples (comprising 507 LUAD samples and 60 normal samples) were selected from the GSE72094 and TCGA-LUAD datasets, respectively. To convert the transcriptome matrix of TCGA-LUAD from fragments per kilobase million (FPKM) to transcripts per million (TPM), the “limma” script was utilized. The “sva” package was employed to address the batch effect and normalize the transcriptome matrix of LUAD samples from both the GSE72094 and TCGA-LUAD datasets. Copy number variation (CNV) at the gene level for LUAD was obtained from the UCSC Xena database.

### Molecular subtype characterization identification

In the present investigation, we have identified 16 genes associated with disulfidptosis (DAG) from previously published research (Supplementary Table 1) [12]. The R script “limma” was employed to extract the expression profiles of these genes. Subsequently, the mutation file of LUAD samples was downloaded from the TCGA-LUAD dataset, and the “maftools” script was utilized to explore the mutation frequency of the 16 DAG. We calculated the hazard ratio (HR) of DAG using the univariate Cox algorithm, and established a network to visualize the relationship between prognosis and DAG using the “igraph” script. We employed the “ConsensusClusterPlus” script to classify LUAD samples into different molecular subtypes based on the expression levels of the 16 DAG, using the best classification of K = 2–9 [13–15]. We evaluated the clinical survival outcome of LUAD samples in DAG-based molecular subtypes using the “survival” script and log-rank method. Principal component analysis (PCA) was utilized to investigate the distribution pattern of DAG-based molecular subtypes using the “ggplot2” script. Finally, we utilized the “pHeatmap” script to visualize the relationship between DAG expression profiles, clinical survival status, and clinical variables.

### Differential expression analysis between DAG molecular subtypes

To determine the differentially expressed genes (DEGs) between the subtypes defined by DAG, we employed the “limma” script and applied a *P*-value (adjusted) threshold of < 0.05 for selection. In order to obtain enriched DEGs, we utilized gene ontology (GO) and Kyoto Encyclopedia of Genes and Genomes (KEGG) assessment algorithms, which were implemented through the “clusterProfiler” script. The DEGs were subsequently used to identify gene-cluster subtypes of LUAD via the “ConsensusClusterPlus” script.

### Development of prognostic signature and independent prognosis analysis

Initially, we conducted LASSO-univariate Cox analysis to identify prognostic variables for LAUD, utilizing a selection filter of *p* < 0.05. Subsequently, based on multivariate Cox analysis, we identified independent characteristic prognostic factors, in order to establish a prognostic signature for LAUD. The DAG score of each LUAD sample was then calculated using the coefficients obtained from multivariate Cox analysis and the DAG expression profile, using the formula: DAG score = G6PD × (0.79) + S100P × (0.40) + CX3CL1 × (−0.47) + EPS8L3 × (0.21) + MS4A15 × (−0.25) + GSTA1 × (−0.48) + KRT6A × (0.27) [13, 14]. By utilizing the “caret” script, we classified the LUAD samples into training and test cohorts, using a division ratio of 7:3, based on the median DAG score, into DAG low- and high-score groups [16]. Finally, we employed the time-dependent ROC curve using the “survivalROC” script to estimate the area under the curve (AUC) for one, three, and five-year survival. The “ComplexHeatmap” package was utilized to demonstrate the variation in DAG score across clinical variables (age, gender, and stage) for LUAD. To estimate the clinical prognosis of LUAD samples under different clinical variables, we used the “survminer” script based on the median DAG score. We developed a nomogram for LUAD with the “rms” script, taking into account the DAG score and clinical features. To assess the prognostic independence of DAG score and clinical variables, we performed univariate and multivariate Cox analysis using the “survival” script.

### Immune-infiltration characteristics, immune therapy response, and tumor mutation features

We applied the ESTIMATE method to assess the immune status of LUAD samples and computed four parameters, including ESTIMATE, immune and stromal scores, and tumor purity. We evaluated the immune infiltration characteristics of LAUD samples by using a gene set for 23 types of immune cells and obtained the relative percentage of each cell type via ssGSEA algorithm [15]. To predict the response of LUAD to CTLA-4 or PD-1 immunotherapy, we utilized the publicly available immunotherapy database TCIA (The Cancer Immunome Atlas, https://tcia.at/home). To predict the response of LUAD to chemotherapy drugs based on DAG score subtypes, we used the “pRRophetic” script and the GDSC database (https://www.cancerrxgene.org/). We obtained the mutation files (maf) of LUAD from the TCGA database and evaluated the gene mutation landscape of LUAD in DAG score subtypes using the “maftools” script.

### qRT- PCR analysis and Ki67 immunostaining

The cells were lysed by using Trizol reagent (Invitrogen, USA; Cat# 15596-026) in order to extract the total RNA. RT-qPCR was performed as per the manufacturer’s instructions. The primer sequences, synthesized by Sangon Biotech (Shanghai) Co., Ltd., were listed in Supplementary Table 2. The amplification conditions involved a temperature of 42°C for 5 minutes, followed by an initial denaturation at 95°C for 10 seconds. This was followed by 40 cycles of denaturation at 95°C for 5 seconds and annealing at 60°C for 31 seconds. The obtained results were analyzed using the 2^−ΔΔCt method with β-actin serving as the internal control reference. The cells were then washed with PBS and fixed with 4% paraformaldehyde for 15 minutes. Subsequently, 500 μL of 1% TritonX-100 solution was added and incubated at room temperature for 30 minutes. After rinsing with PBS, the cells were incubated with Ki67 antibody (1:200) overnight at 4°C, followed by incubation with Alexa Fluor 594-conjugated secondary antibody (1:200) at room temperature for 1 hour. After washing with PBS, 5 μL of DAPI working solution was added and incubated at room temperature for 5 minutes. Finally, the cells were observed and photographed with the aid of an Olympus FV1000 microscope.

### Cell culture and western blot

The A549 human lung cancer cell line and the BEAS-2B normal bronchial epithelial cell line were obtained from the Shanghai Cell Bank of the Chinese Academy of Sciences. The cells were cultured in complete RPMI-1640 medium supplemented with 10% fetal bovine serum, 100 U/mL penicillin, and 100 mg/L streptomycin, and passaged in a humidified atmosphere containing 5% CO2 at 37°C. Total protein was extracted from the cells using RIPA buffer. The protein concentration was determined using the BCA method, and then subjected to SDS-PAGE electrophoresis, followed by transfer, blotting, and blocking. The membranes were probed overnight at 4°C with a rabbit anti-human G6PD antibody (1:1000) (Cat#8866S, CST, USA). After washing three times with TBST buffer, the membranes were incubated with the secondary antibody (1:20000) at room temperature for 1 hour. The membranes were then washed three times with TBST buffer, imaged using Odyssey Clx (Li-Cor, USA), and analyzed for grayscale values using Image J software. The internal control reference was β-actin.

### Cell cloning analysis

The cell line A549 in the logarithmic phase was seeded in a 6-well cell culture plate at a density of 800 cells per well. After an overnight incubation, the experimental group was treated with G6PD inhibitor G6PDi (0.05 μM) (HY-W107464, MCE, USA) [17], while the control group received an equivalent volume of dimethyl sulfoxide (DMSO) solution. The cells were cultured for 14 days and then stained with crystal violet solution and photographed.

### Statistics analysis

The data for LUAD was obtained and processed using R (×64, 4.1.0) and Perl programming languages. Differences between two groups were assessed using the Wilcoxon test, while statistical significance among three groups was evaluated using ANOVA. The correlation between DAG score and immune infiltration was analyzed using Spearman correlation analysis. The log-rank algorithm was employed to compare clinical survival outcomes among different groups. A significance level of *P* < 0.05 was considered for all comparisons between groups.

## RESULTS

### Expression profile, CNV and somatic mutation identification of disulfidptosis-associated genes (DAG) in LUAD

**Figure 1:**
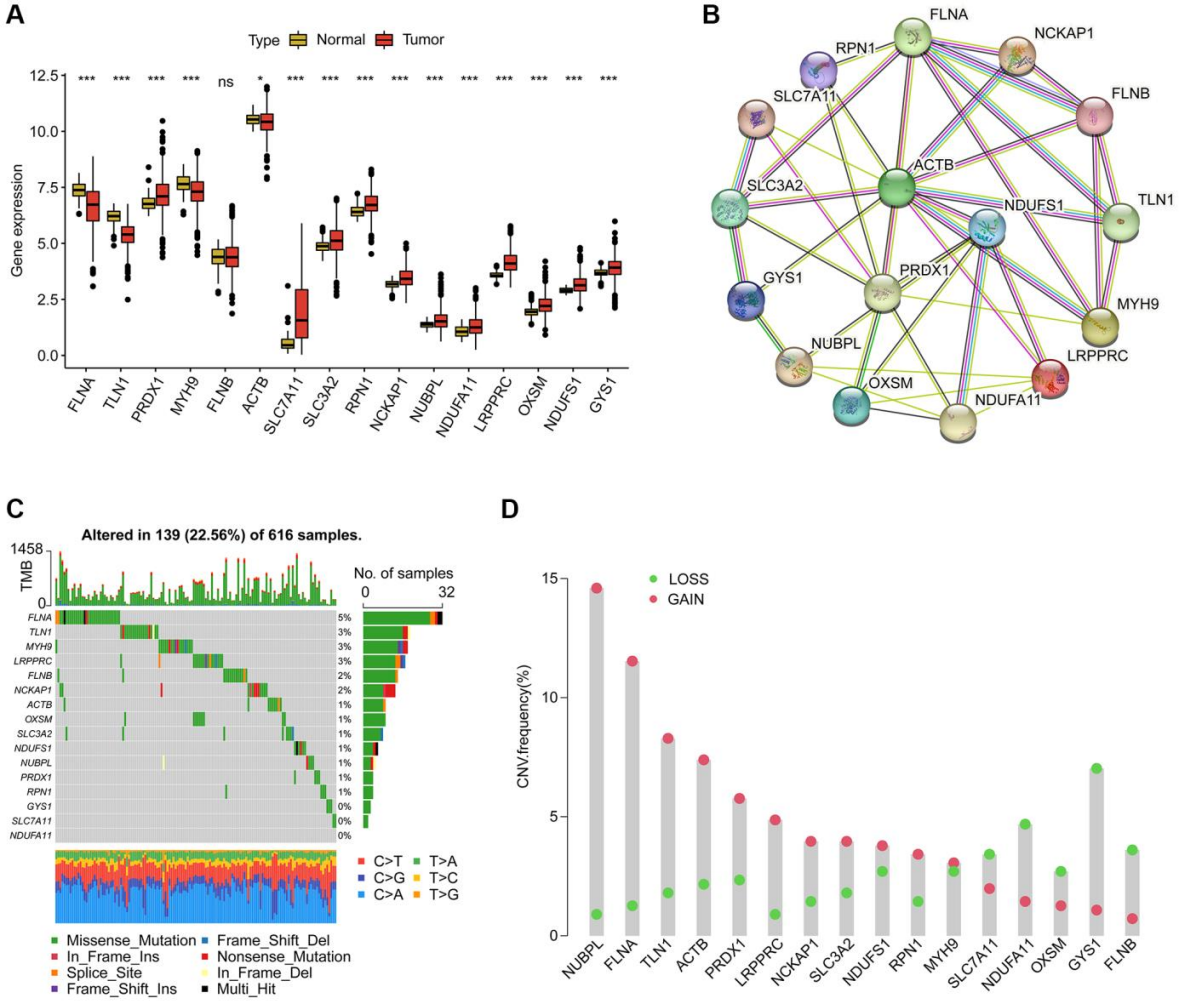
**Analysis of disulfidptosis-associated genes (DAG) in LUAD.** (**A**) Differential analysis of DAG in normal and LUAD tissues. (**B**) Interaction analysis of 16 DAG. (**C**) Estimation of somatic mutation of DAG in LUAD. (**D**) Evaluation of CNV frequency of DAG.

We identified 16 disulfidptosis-associated genes (DAG) and investigated their potential function in the progression of LUAD. Differential expression analysis revealed distinct expression profiles of these genes between normal and tumor tissues (Figure 1A). Specifically, FLNA, TLN1, MYH9, and ACTB were down-regulated in tumor tissues, while PRDX1, SLC7A11, SLC3A2, RPN1, NCKAP1, NUBPL, NDUFA11, LRPPRC, OXSM, NDUFS1, and GYS1 were overexpressed. The PPI network analysis suggested potential interactions among these genes (Figure 1B). Somatic mutation analysis showed that 139 LUAD samples (22.56%) had mutation frequency variation in DAG out of 616 samples, with FLNB, LRPPRC, MYH9, and TLN1 having mutation frequencies of 6%, 3%, 3%, and 3%, respectively (Figure 1C). In addition, the CNV analysis revealed CNV amplification of NUBPL, FLNA, TLN1, ACTB, PRDX1, LRPPRC, NCKAP1, SLC3A2, NDUFS1, and RPN1, while SLC7A11, NDUFA11, OXSM, GYS1, and FLNB showed higher CNV deletion (Figure 1D). These findings indicate a differential landscape of DAG expression profile, mutation, and CNV, suggesting their potential role in the carcinogenesis of LUAD.

### Characterization of DAG-based molecular subtypes in LUAD

**Figure 2:**
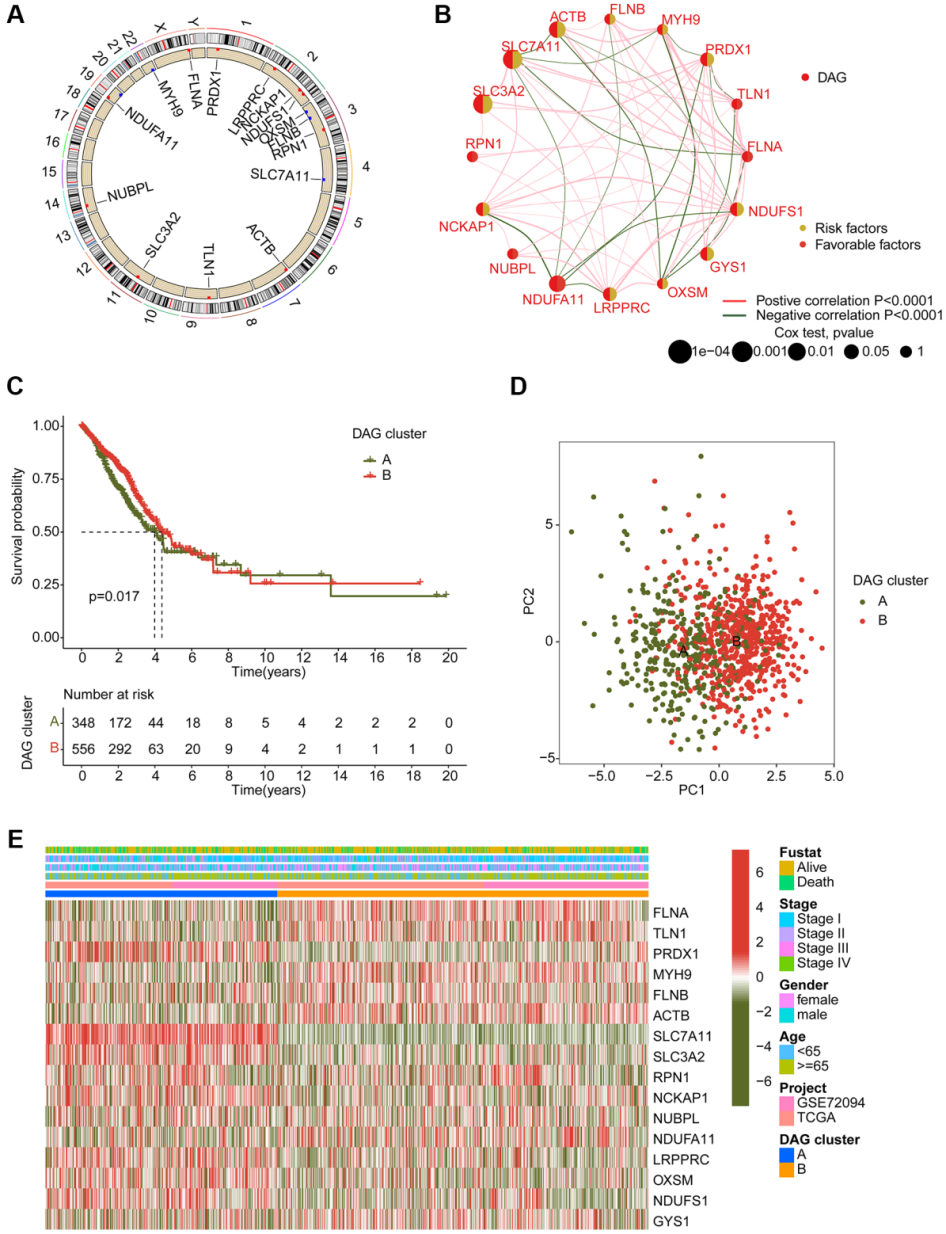
**Generation of DAG-based molecular subtypes.** (**A**) Location of DAG in chromosome. (**B**) Analysis of correlation and prognostic value of DAG in LUAD. (**C**) Clinical survival prognosis curve. (**D**) Unsupervised PCA of LUAD samples in DAG-based molecular subtypes. (**E**) Relationship of DAG expression and clinical variables (survival status, age, stage and gender) in DAG-based molecular subtypes.

In order to determine the DAG-based molecular subtypes of LAUD, we collected 397 LAUD samples from GSE72094 and 507 LAUD samples from the TCGA-LUAD dataset. As depicted in Figure 2A, a circular plot was used to illustrate the location of the 16 DAGs in the chromosome. The network plot was used to demonstrate the relationship and prognostic value of the DAGs in LAUD. 9 prognostic variables were obtained through univariate Cox analysis, including a favorable factor (NDUFA11) and 8 risk factors (NCKAP1, PRDX1, NDUFS1, LRPPRC, GYS1, ACTB, SLC7A11, and SLC3A2) (Figure 2B). Based on the expression profile of the 16 DAGs, two molecular subtypes of LAUD samples were successfully identified using an unsupervised consensus clustering algorithm. Notably, a significant difference in clinical prognosis was observed between the two DAG-based molecular subtypes, with the clinical prognosis of LAUD samples in DAG-cluster A being worse compared to those in DAG-cluster B (*p* = 0.017, Figure 2C). Furthermore, the principal component analysis (PCA) diagram could clearly differentiate the LAUD samples in DAG-cluster A and B into different distribution patterns, indicating the independence of the DAG-based molecular subtypes (Figure 2D). The heatmap showed the relationship between the expression of the 16 DAGs and clinical variables (survival status, age, gender, and stage) in DAG-cluster A and B. A distinct difference was observed in the expression profiles of the 16 DAGs (Figure 2E).

### Immune infiltration estimation of DAG-based molecular subtypes

**Figure 3:**
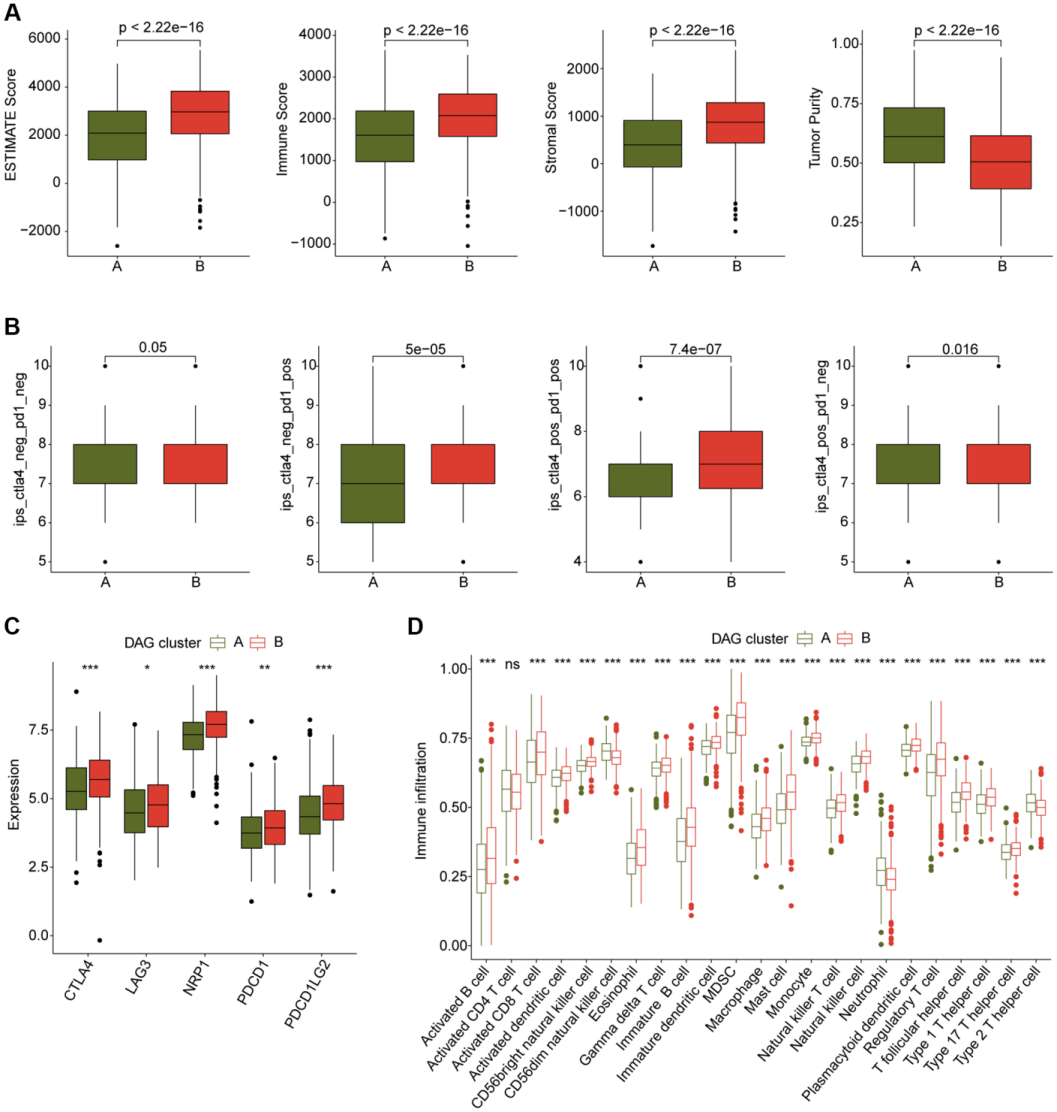
**Immune microenvironment landscape characteristic in DAG-based molecular subtypes.** (**A**) ESTIMATE score assessment in DAG-based subtypes. (**B**) Immunotherapy response of LUAD samples in DAG-cluster A and B. (**C**) Expression of immune checkpoints in DAG-based subtypes. (**D**) Estimation of immune cells.

We also conducted an analysis of immune infiltration and immunotherapy response in the DAG-based molecular subtypes of LUAD samples. The results of immune status estimation revealed that LUAD samples with better clinical survival prognosis in DAG-cluster B had higher immune status and lower tumor purity (Figure 3A). Furthermore, the results of immunotherapy response assessment suggested that LUAD samples in DAG-cluster B were more responsive to PD-1 or CTLA-4 treatment strategies (Figure 3B). The immune checkpoint result indicated that the expression of CTLA-4, LAG3, NRP1, PDCD1, and PDCD1LG2 was significantly higher in LUAD samples in DAG-cluster B (Figure 3C). Additionally, the ssGSEA assessment revealed a significantly higher proportion of most immune cells in LUAD samples in DAG-cluster B, indicating a higher immune infiltration status of the samples in DAG-cluster B (Figure 3D). Overall, our findings demonstrate a significant difference in the immune microenvironment between the DAG-based molecular subtypes of LUAD, which is related to the immunotherapy response.

### Comprehensive analysis of DAG-related DEGs in LUAD

**Figure 4:**
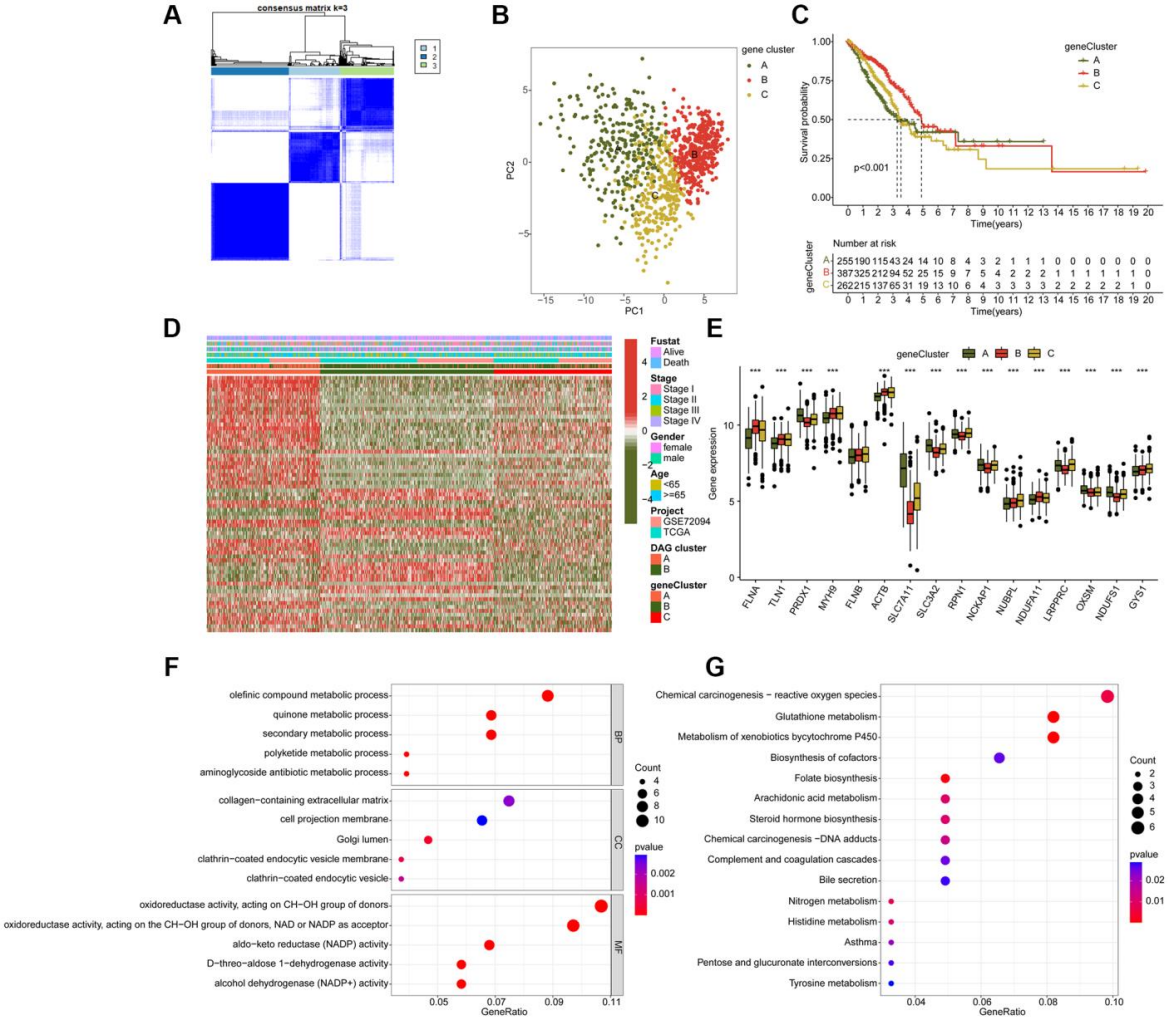
**Molecular characteristic of DAG-related gene-cluster subtypes in LUAD.** (**A**) Identification of DAG-related gene-cluster subtypes. (**B**) Unsupervised PCA analysis of gene-cluster A, B and C. (**C**) Clinical prognosis analysis of LUAD in gene-cluster A, B, and C. (**D**) Comprehensive analysis of DAG-related DEGs in gene-cluster, DAG cluster and clinical variables. (**E**) The expression of DAG in gene-cluster A, B and C. (**F**, **G**) GO and KEGG enrichment analysis of DAG-related DEGs.

To further investigate the biological function of DAG subtypes in LUAD, we conducted a differential analysis to identify differentially expressed genes (DEGs) among the DAG-based subtypes using the “limma” script. A total of 107 DEGs were identified for the consensus clustering analysis, and three gene cluster subtypes were explored, with 255 samples in gene cluster A, 387 samples in gene cluster B, and 262 samples in gene cluster C (Figure 4A). The PCA plot also demonstrated an independent division pattern of LUAD samples in gene cluster subtypes (Figure 4B). Clinical outcome analysis indicated a better prognostic probability of LUAD samples in gene cluster B compared to gene cluster A and C (*p* < 0.001, Figure 4C). As shown in Figure 4D, the heatmap diagram illustrates the relationship between DEGs expression and clinicopathological features. Furthermore, we also observed a significant difference in DAG expression among gene cluster subtypes, such as FLNA, TLN1, PRDX1, and MYH9 (Figure 4E). GO enrichment analysis suggested that the DEGs were involved in metabolism-related biological functions, such as olefinic compound metabolic process, quinone metabolic process, and secondary metabolic process (Figure 4F). Additionally, the KEGG result demonstrated that the DEGs were enriched in chemical carcinogenesis-reactive oxygen species, glutathione metabolism, and metabolism of xenobiotics by cytochrome P450 (Figure 4G).

### Construction of prognostic signature based on DAG-related DEGs

**Figure 5:**
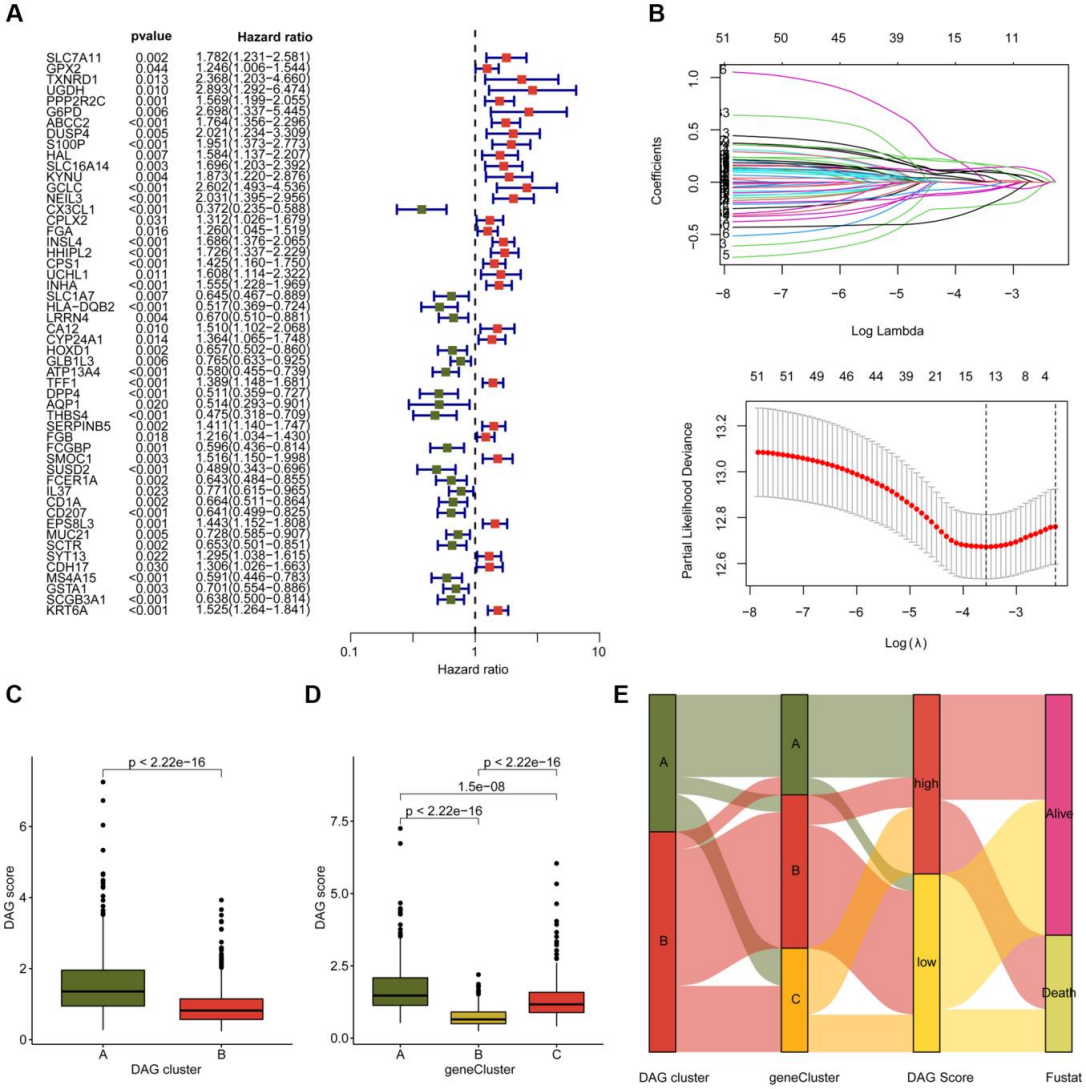
**Development of DAG score based on the prognostic DAG-related DEGs.** (**A**) The univariate Cox analysis of DAG-related DEGs. (**B**) Identification of feature prognostic variables via LASSO analysis. (**C**) Difference analysis of DAG score in DAG cluster subtypes. (**D**) Distribution of DAG score in gene cluster A, B and C. (**E**) Alluvial plot shows the potential relationship of DAG score and clinical survival outcome in DAG cluster subtypes and gene cluster subtypes.

We conducted an analysis to evaluate the prognostic value of DAG-related DEGs for LUAD. Specifically, we performed univariate Cox analysis and identified 52 prognostic factors with *p* < 0.05, including 21 favorable factors and 31 risk factors (Figure 5A). Using the “glmnet” package, we further conducted LASSO analysis and selected 16 vital variables from the 52 prognostic factors (Figure 5B). Based on the multivariate Cox analysis, we established a prognostic risk model consisting of 7 independent prognostic factors and calculated the DAG score of LUAD samples. In the DAG cluster subtypes, we observed that LUAD samples with a better clinical prognosis in DAG-cluster B had a lower DAG score (Figure 5C). Similarly, in the gene cluster subtypes, we found that LUAD samples with longer survival prognosis had a lower DAG score, suggesting a potential association between DAG score and clinical outcome for LUAD samples (Figure 5D). The alluvial diagram illustrated the potential relationship between DAG score and clinical prognosis in DAG cluster subtypes and gene cluster subtypes (Figure 5E). Specifically, LUAD samples with favorable clinical survival outcome tended to divide into the low DAG score group and were associated with better survival outcome.

### Development and verification of DAG prognostic signature

**Figure 6:**
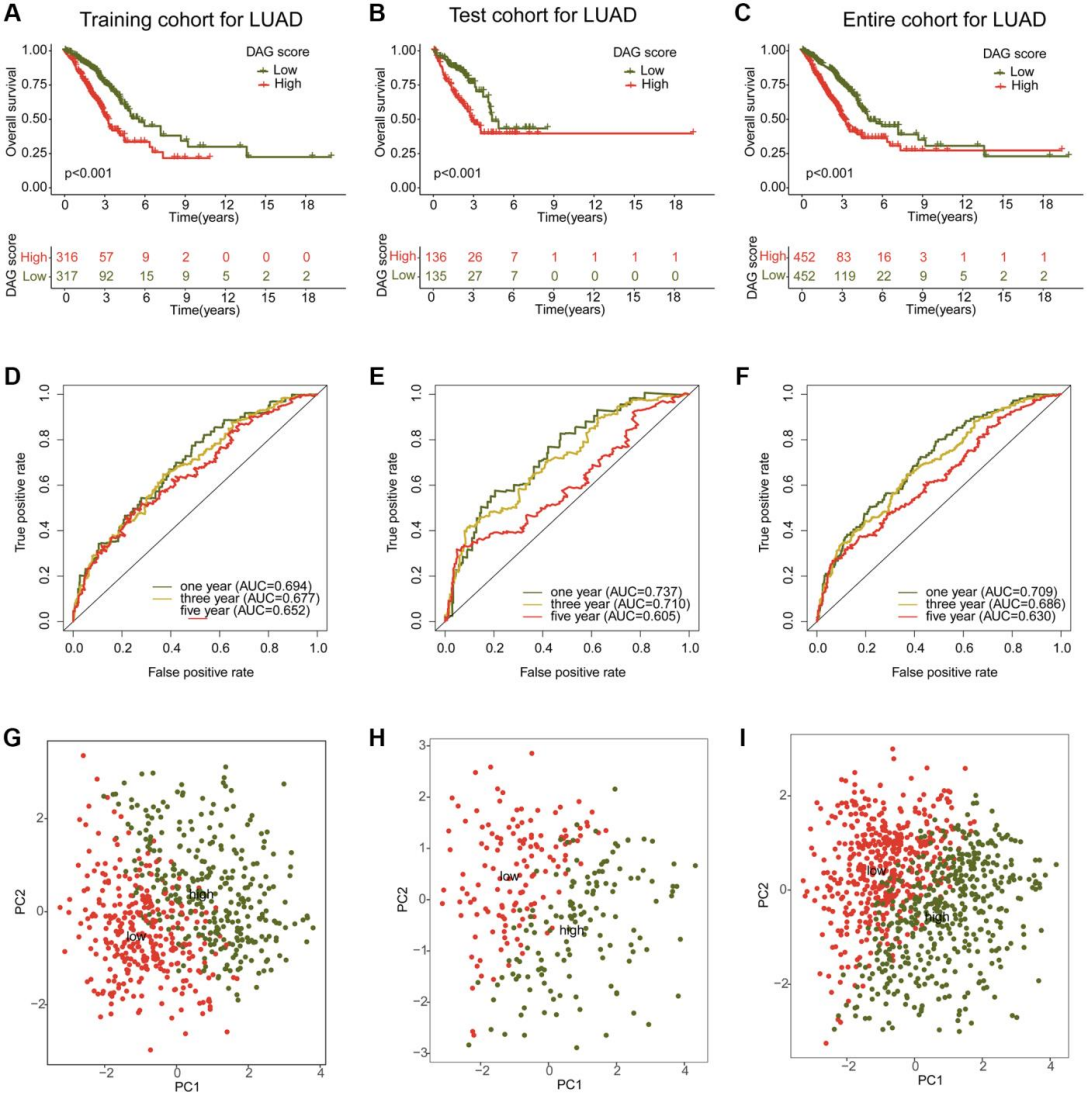
**Construction and validation of risk model based on the DAG prognostic signature.** (**A**–**C**) Clinical survival curve analysis of LAUD samples in the risk subtypes in the training, test and entire cohorts. (**D**–**F**) One- three- and five-year’s AUC in the training, test and entire cohorts. (**G**–**I**) PCA diagram of LUAD samples with low- and high DAG score in the training, test and entire cohorts.

To investigate the potential association between the DAG score and clinical outcome prognosis for LUAD samples, the samples were divided into a training cohort and a test cohort in a 7:3 ratio using the “caret” script. The samples were then categorized as low- or high-DAG score cohorts based on the median DAG score in the training and test cohorts. The clinical outcomes of the LUAD samples in both independent cohorts demonstrated that those with low DAG scores had significantly better prognoses than those with high DAG scores (Figure 6A, 6B). Furthermore, the clinical survival outcomes of the entire cohort revealed that the low DAG score group had a better survival outcome than the high DAG score group (Figure 6C). The AUC of the ROC curve at one, three, and five years was 0.693, 0.677, and 0.652 in the training cohort; 0.737, 0.710, and 0.605 in the test cohort; and 0.709, 0.686, and 0.630 in the entire cohorts (Figure 6D–6F). Additionally, the PCA diagram clearly distinguished the LUAD samples with low and high DAG scores into two distinct distribution patterns in the training, test, and entire cohorts (Figure 6G–6I). These findings demonstrate that the DAG prognostic signature can accurately classify LUAD samples into different risk subtypes and is associated with clinical survival outcomes.

### Integrated analysis of DAG score in different clinicopathological characteristics

**Figure 7:**
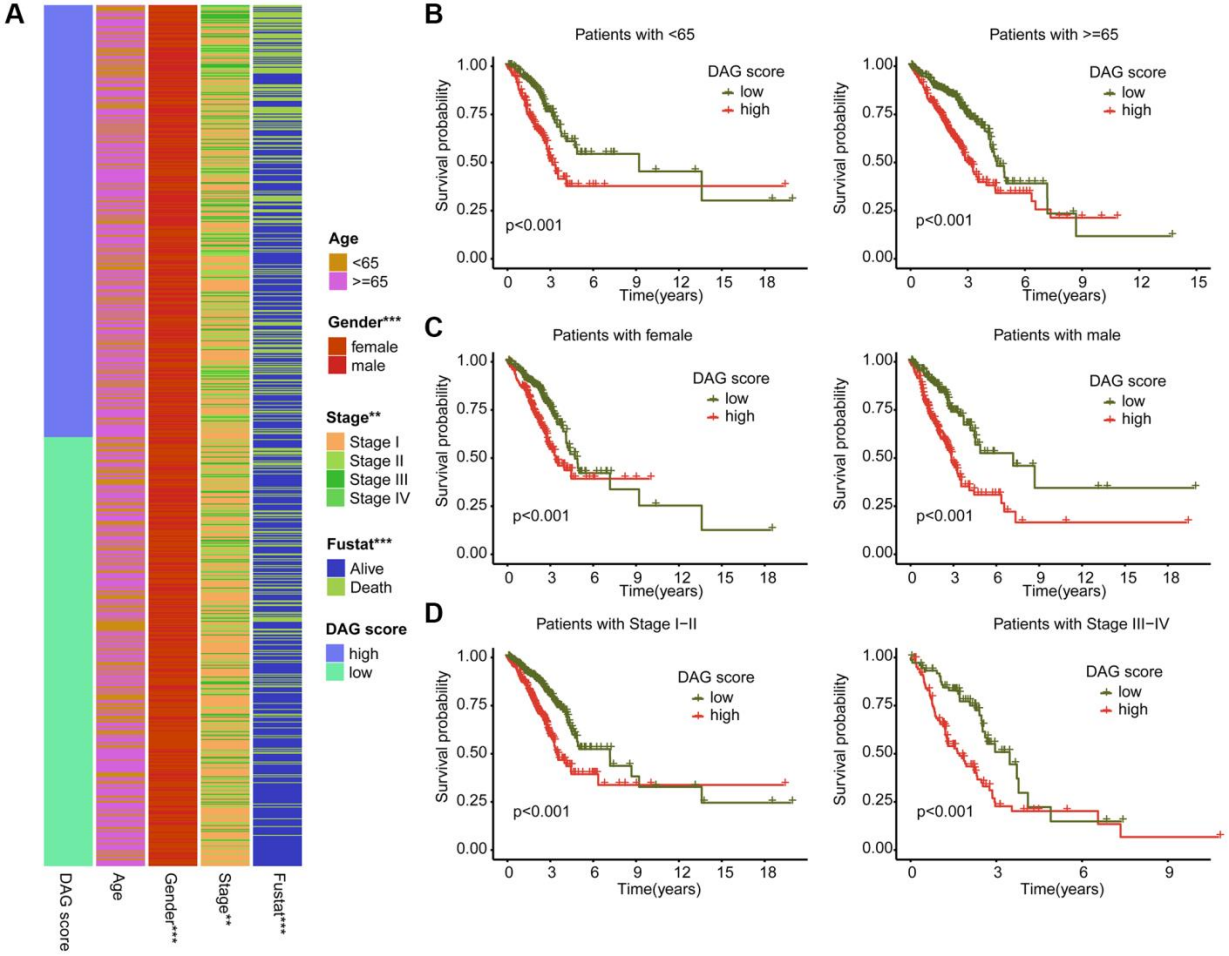
**Association analysis of DAG score and clinical variables.** (**A**) Heatmap diagram shows the DAG score distribution in the different clinicopathological characteristics. (**B**–**D**) Clinical prognosis analysis of LUAD samples with low- and high DAG score among age, gender and stage.

We further examined the role of DAG score in relation to various clinical clinicopathological characteristics. As shown in Figure 7A, a significant difference in DAG score was observed in relation to gender, stage, and clinical survival status. Subsequently, we evaluated the prognostic value of DAG score in different clinical variables, and found that the survival outcome of LUAD samples with high DAG score was worse compared to those samples with low DAG score among different age, gender, and stage groups (Figure 7B–7D).

### Nomogram development and independent prognosis analysis of DAG score

**Figure 8:**
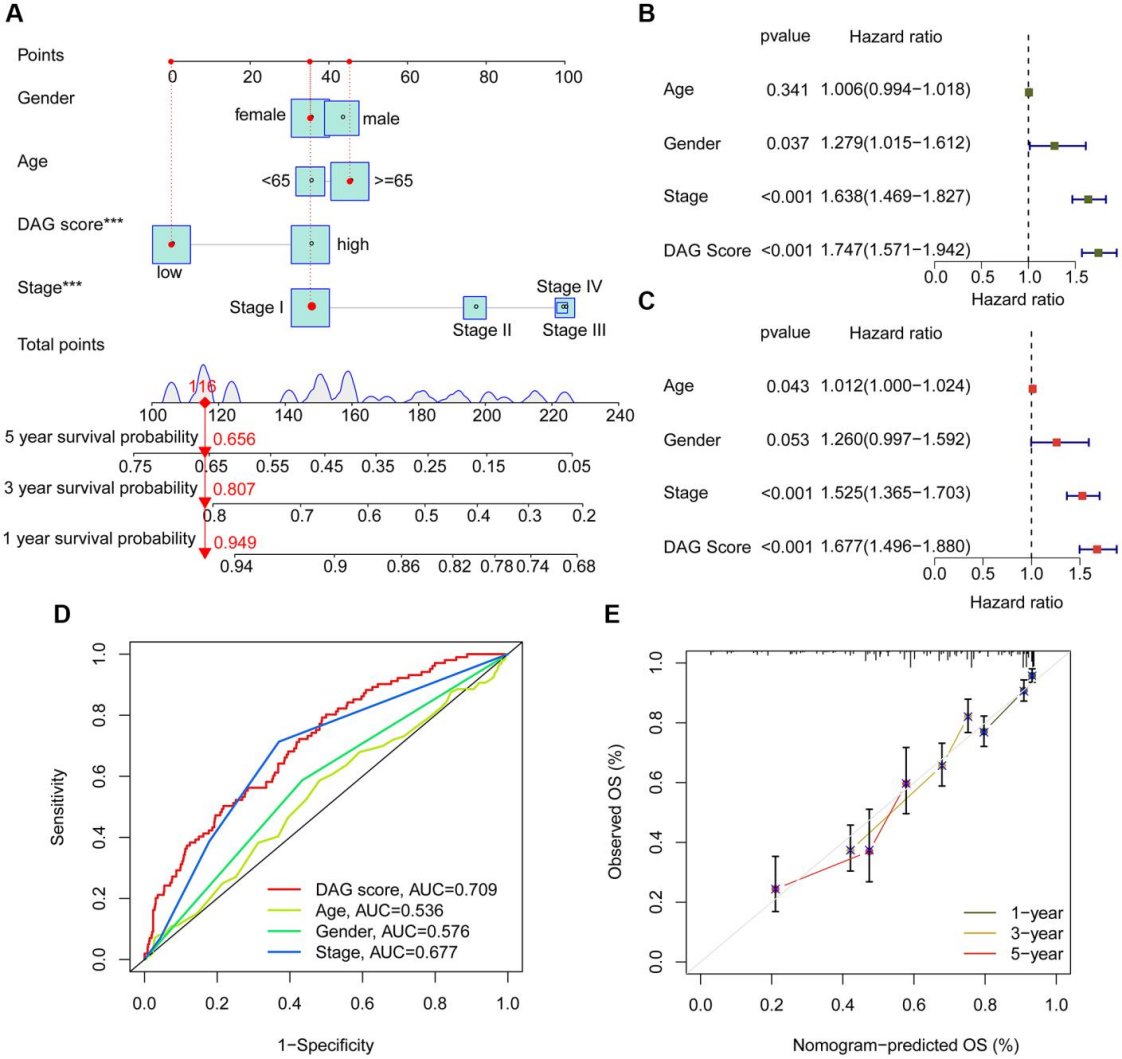
**Nomogram establishment and independent prognosis analysis.** (**A**) Nomogram construction of DAG score and clinical variables. (**B**, **C**) The analysis of univariate and multivariate Cox analysis to evaluate independence of DAG score and clinicopathological features. (**D**) ROC curve analysis shows the AUC of DAG score, age, gender and stage. (**E**) Correction curve for survival prediction.

Using integrated clinical information from the TCGA-LUAD and GSE72094 datasets, we developed a nomogram to explore the one, three, and five-year survival ability of LUAD samples based on DAG score and clinical variables (Figure 8A). Univariate Cox analysis revealed that gender (HR = 1.279, 95% CI = 1.015–1.612, *P* = 0.037), stage (HR = 1.638, 95% CI = 1.469–1.827, *P* < 0.001), and DAG score (HR = 1.747, 95% CI = 1.571–1.942, *P* < 0.001) were considered as risk factors for LUAD (Figure 8B). Multivariate Cox analysis showed that stage (HR = 1.525, 95% CI = 1.365–1.703, *P* < 0.001) and DAG score (HR = 1.677, 95% CI = 1.496–1.880, *P* < 0.001) were independent prognostic predictors for LUAD (Figure 8C). The ROC curve displayed that the area under the curve (AUC) of DAG score, age, gender, and stage was 0.709, 0.536, 0.576, and 0.677, respectively, suggesting a favorable diagnostic ability of DAG score for LUAD (Figure 8D). Moreover, the calibration curve of one, three, and five-year showed optimal consistency between actual survival probability and nomogram-predicted survival probability (Figure 8E).

### Estimation of immune infiltration and immunotherapy response in DAG score subtypes

**Figure 9:**
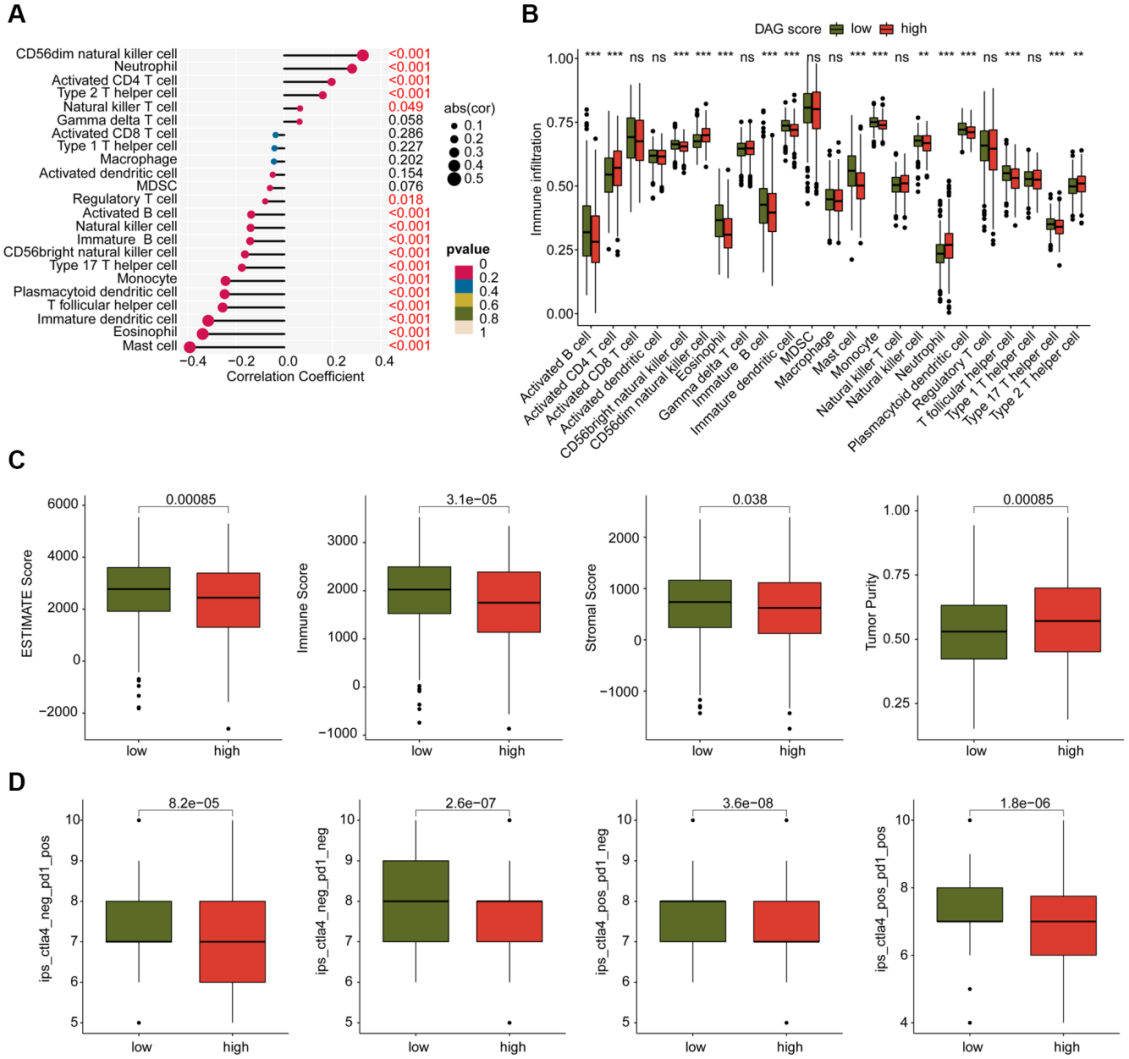
**Analysis of immune infiltration and immunotherapy response in DAG score subtypes.** (**A**) Correlation analysis of DAG score and immune infiltration. (**B**) The proportion of 23 immune cells in the DAG score subtypes. (**C**) Immune status estimation. (**D**) Immunotherapy response analysis of DAG score subtypes.

We conducted an investigation into the relationship between DAG score and immune infiltration. As shown in Figure 9A, we observed a significant correlation between DAG score and immune infiltration. Specifically, the DAG score was positively associated with CD56dim natural killer cells, neutrophils, CD4^+^ T cells, and type 2 T helper cells, while it was negatively associated with mast cells, eosinophils, immature dendritic cells, T follicular helper cells, and plasmacytoid dendritic cells. Our analysis of ssGSEA results revealed that the proportion of most immune cells was lower in the high DAG score group than in the low DAG score group, indicating an immunosuppressed status of LUAD samples with high DAG score (Figure 9B). Additionally, based on the ESTIMATE algorithm, the high DAG score group had lower scores for ESTIMATE, immune and stromal components, and higher tumor purity (Figure 9C). Furthermore, we evaluated the immunotherapy response of LUAD samples in different DAG score subtypes using the TCIA database, and found that the samples with low DAG score were more responsive to the PD-1 and CTLA-4 treatment strategies (Figure 9D). These findings suggest a potential association between DAG score and immune infiltration, and highlight the possibility of predicting immunotherapy response based on DAG score subtypes in LUAD.

### Chemotherapeutic drugs evaluation and tumor mutation burden characteristic

**Figure 10:**
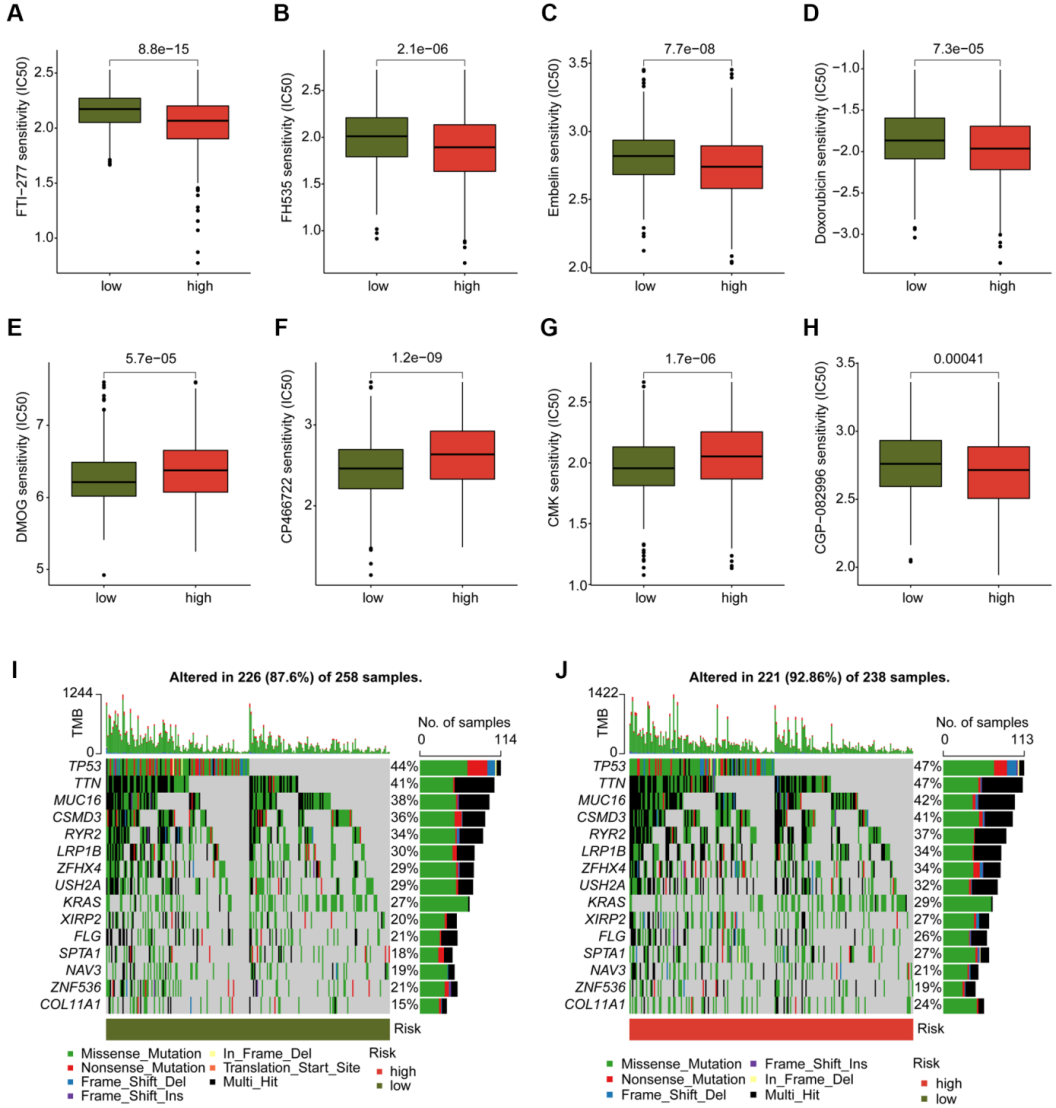
**Analysis of chemotherapeutic drugs sensitivity and tumor mutation burden landscape in DAG score subtypes.** (**A**–**H**) Chemotherapeutic drugs sensitivity analysis of FTI-277, FH535, Embelin, Doxorubicin, CGP-082996, DMOG, CP466722 and CMK in DAG score subtypes. (**I**, **J**) Somatic mutation frequency analysis of DAG score subtypes.

We conducted an evaluation of several chemotherapeutic drugs that may be beneficial for the treatment of LUAD across different DAG score subtypes. As depicted in Figure 10A–10H, we observed that LUAD samples with high DAG score had a lower IC50 value for FTI-277, FH535, Embelin, Doxorubicin, and CGP-082996, while the IC50 value of DMOG, CP466722, and CMK was higher in the DAG score for LUAD. Additionally, we explored the TMB characteristics of LUAD samples in DAG score subtypes. We found that 226 samples (87.6%) of 258 LUAD samples had somatic mutations in the low DAG score group, while 221 samples (92.86%) of 238 LUAD samples had somatic mutations in the high DAG score group (Figure 10I, 10J). Our mutation frequency results suggested that LUAD samples with high DAG score had a higher somatic mutation frequency, including TP53 (47%), TTN (47%), MUC16 (42%), and CSMD3 (41%).

### Downregulation of G6PD can inhibit the proliferation and cloning of lung cancer cells

**Figure 11:**
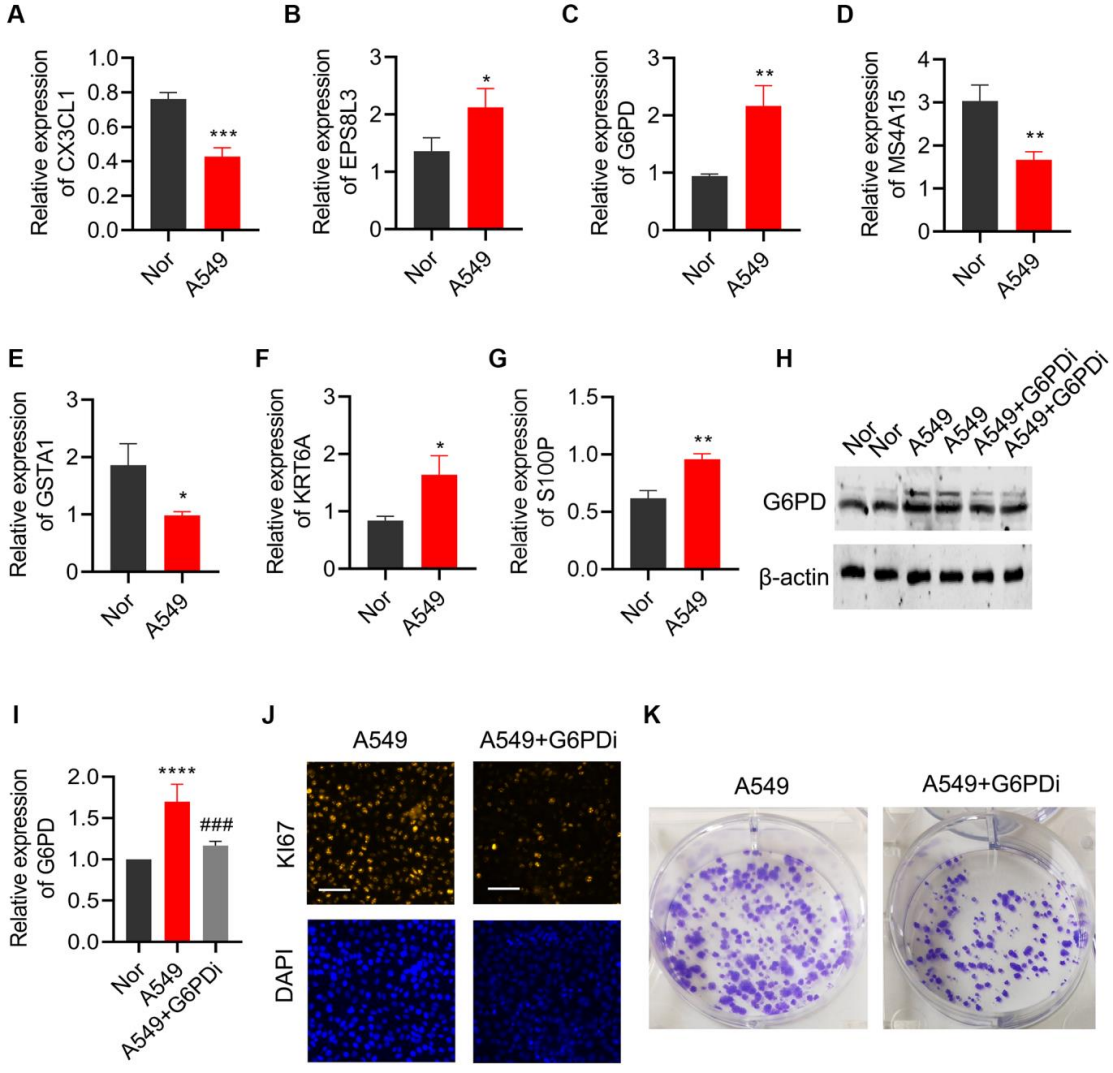
**Effects of regulating G6PD on the proliferation and colony formation of lung cancer cells.** The levels of (**A**) CX3CL1, (**B**) MS4A15, (**C**) GSTA1, (**D**) EPS8L3, (**E**) G6PD, (**F**) KRT6A, and (**G**) S100P were detected by qPCR in BEAS-2B and A549 cells. (**H**, **I**) G6PD expression in A549 cells was analyzed by Western blot after treatment with G6PDi. (**J**) Ki67 staining was used to assess the proliferation ability of cells after interfering with G6PD. (**K**) Colony formation assay was performed to detect the proliferation ability of cells after interfering with G6PD.

qPCR was utilized to validate the expression of the 7 key genes that were screened in both lung cancer cell line (A549) and normal bronchial epithelial cell line (BEAS-2B). Results indicated a significant downregulation of CX3CL1, MS4A15, and GSTA1 in lung cancer cells, while EPS8L3, G6PD, KRT6A, and S100P were notably upregulated, in line with the bioinformatics analysis findings (Figure 11A–11G). As G6PD showed the highest risk coefficient for lung cancer, the impact of G6PD downregulation on lung cancer cell proliferation and colony formation was investigated. Western Blot analysis exhibited a significant increase in G6PD expression in A549 cells compared to the BEAS-2B group, which was consistent with the qPCR results. Treatment with G6PD inhibitor G6PDi effectively reduced G6PD expression in lung cancer cells (Figure 11H, 11I). Results from Ki67 staining and colony formation assays demonstrated that interfering with G6PD can substantially inhibit the proliferation ability of lung cancer cells (Figure 11J, 11K).

## DISCUSSION

In this study, we verified the validity of subtype analysis based on disulfidptosis genes and construction of a prognostic risk model in LUAD patients. qPCR and WB were also used to verify the significant abnormal expression of selected targets in lung cancer cells and the anti-tumor effect on the proliferation of lung cancer cells, suggesting a potential role of disulfidptosis in LUAD. Although there is limited evidence of a role of disulfidptosis in tumors, clues related to disulfide stress suggest a role and clinical value of disulfidptosis in tumors.

Glucose starvation induced apoptosis in cancer cells with relatively high SLC7A11 expression level [18]. Treatment to prevent disulfide accumulation inhibited cell death with high SLC7A11 expression [19]. The above evidence shows a correlation between disulfide stress and cell death. Our data show the accuracy of disulfidptosis related risk stratification in predicting the prognosis of LUAD and the positive significance of related genes in LUAD prognosis. High levels of SLC7A11 in LUAD and its association with prognosis have been demonstrated [8]. There is a number of evidences suggesting the role of disulfidptosis in lung cancer, including LUAD. Protein disulfide isomerase (PDI) preserves the natural conformation and stability of other proteins through disulfide bond formation, isomerization, and rearrangement [20]. PDI is highly expressed in a variety of tumors and is a potential therapeutic target [21]. The expression level of PDI was increased in lung cancer, and its members PDIA4 and PDIA6 were up-regulated in response to cisplatin treatment [22, 23]. At the same time, PDI inactivation directly stimulated lung cancer cell death through different cell signaling pathways [23]. In addition, disulfide reductase inhibitors have been reported to induce oxidative stress and lethality in lung cancer cells [24]. The reason why these disulfide bond formation related proteins lead to cell death and whether the mechanism as a potential therapeutic target is due to glucose starvation leading to the rapid accumulation of disulfide bond molecules in LUAD cells remains to be further investigated.

Among the selected prognostic markers, G6PD showed maximum risk coefficient as a pro-carcinogenesis factor. G6PD (Glucose-6-phosphate dehydrogenase) is an enzyme that plays a key role in glucose metabolism [25]. Treatment with G6PD inhibitor effectively reduced G6PD expression in lung cancer cells and substantially inhibited the proliferation ability of lung cancer cells, which showed the first time of the adverse prognostic significance and pro-carcinogenesis effect of G6PD in LUAD. In addition to promoting cancer cell growth and survival by providing the energy needed for cancer cell proliferation, G6PD’s role in cancer metabolic reprogramming and REDOX signaling was further demonstrated for its role in cancer progression [26]. G6PD is the largest contributor to NADPH production [27], while NADPH promotes tumor resistance to ROS and ferroptosis [28]. In addition, the expression of cytochrome P450 REDOX reductase, a positive regulator of ferroptosis, was regulated by G6PD [29]. Therefore, G6PD may modulate ferroptosis in a NADPH-dependent manner. Similarly, NAPDH production also affects disulfidptosis processes. A decrease in intracellular NADPH, which leads to actin-skeleton protein disulfide bond formation and F-actin contraction, is one of the important steps in disulfidptosis [5]. Whether the observed association between higher G6PD levels and higher risk levels in LUAD patients is due to differences in resistance to disulfidptosis requires further investigation.

Increased mRNA expression of CX3CL1 has been reported as a positive prognostic factor in patients with lung adenocarcinoma [30]. CX3CL1, a chemokine protein involved in immune response and inflammation, plays a role in recruitment of immune cells to sites of inflammation [31]. We also demonstrated that CX3CL1 was highly expressed in low-risk patients and had a better prognosis. Recent reports suggest that the anti-PD1 immunotherapy effect has also been reported to be related to CX3CL1 mediating, which may be one of the reasons for its influence on LUAD prognosis [32]. S100P plays a crucial role in cancer cell survival and chemotherapy resistance. It promotes lung cancer tumor growth by activating key signaling pathways such as MAPK and PI3K/Akt pathways, thereby participating in cell proliferation and migration [33]. S100P also increases cancer cell resistance to chemotherapy by inhibiting apoptosis [34]. EPS8L3 is involved in the regulation of various cellular processes, including cell growth, proliferation and survival [35]. Although not reported in LUAD, its upregulation is associated with tumorigenesis and poor prognosis in patients with liver cancer, and can be used as a potential therapeutic target [36, 37].

As an endoplasmic reticulum transmembrane protein, MS4A15 regulates ferroptosis balance by improving ferroptosis resistance by calcium-restricted lipid remodeling. Given the important role of ferroptosis in tumor [38], its regulation may be one of the reasons for the effect of MS4A15 on LUAD. Our results show that lower GSTA1 expression levels in high-risk patients are associated with poorer outcomes. GSTA1 is involved in the metabolism of a variety of endogenous and exogenous compounds, including carcinogens, drugs and environmental toxins [39]. The regulation of chemotherapy drug metabolism may be the reason of its influence on prognosis. Evidence has shown carcinogenic effects of KRT6A in lung cancer. KRT6A regulates the pentose phosphate pathway through the MYC pathway, thereby promoting lung cancer cell growth and invasion [40]. In addition, KRT6A promoted EMT and cancer stem cell transformation in lung adenocarcinoma, which is in line with our observation [41].

Our results showed that patients with a higher DAG score had higher infiltrating levels of activated CD4 T cells, neutrophil, CD56 dim NK cells, and lower levels of eosinophils, B cells, mast cell and T follicular helper cell (TfH cell). In these immune cells, a large retrospective study showed no significant correlation between CD56 and LUAD prognosis [42]. In addition, we noted an association between lower levels of TfH cells and poorer prognosis in LUAD. TfH cells are critical for germinal center formation. Elevated TfH level is considered to be an early event in the development of LUAD [43]. TfH cell transcriptional signature enrichment has been reported to be associated with better outcomes in LUAD patients [44]. Further mechanistic studies showed that in the mouse LUAD model, TfH cells produce IL-21, which is essential for tumor control and CD8^+^ T cell effector function in tumor invasion [44]. Our results also support the positive effect of TfH cells on the prognosis of LUAD patients.

In conclusion, we performed cluster typing based on disulfidptosis genes and analyzed differential genes of disulfidptosis subtypes. Seven DAGs were subsequently used to construct a prognostic risk model, and the predictive value of this model for prognosis of LUAD patients was verified. The purpose of our study was to further explore the prognostic markers of LUAD. Together with other studies, the expanded biomarkers library could be further applied for specific gene subtypes screening. LUAD subtypes with specific biomarkers have been shown to help find clinically relevant vulnerabilities [45]. In addition, further explorations of the molecular mechanism of selected biomarkers such as G6PD in the future will help deepen the understanding of the pathogenesis of LUAD.

## Supplementary Materials

Supplementary Tables
