# Supplementary material for: The role of molecular subtypes and immune infiltration characteristics based on disulfidptosis-associated genes in lung adenocarcinoma
Source: Aging (Albany NY). 2023 Jun 13;15(11):5075–95. doi: 10.18632/aging.204782 (PMC10292876; doi:10.18632/aging.204782)
Supplement: Supplementary Tables [file aging-15-204782-s001.pdf]

SUPPLEMENTARY TABLES

Supplementary Table 1. The gene list of disulfidptosis-associated genes (DAGs).

Gene
FLNA
TLN1
PRDX1
MYH9
FLNB
ACTB
SLC7A11
SLC3A2
RPN1
NCKAP1
NUBPL
NDUFA11
LRPPRC
OXSM
NDUFS1
GYS1

Supplementary Table 2. Primer sequences.

G6PD	F	GACGACGAAGCGCAGACA
	R	TCCGACTGATGGAAGGCATC
S100P	F	GAGACAGCCATGGGCATGAT
	R	CGTCCAGGTCCTTGAGCAAT
CX3CL1	F	CTCCGATATCTCTGTCGTGGC
	R	TGTCTCGTCTCCAAGCAGCC
EPS8L3	F	CTCCATCCTGTCCATCACCG
	R	AGTCGAGGTCTGCTTTGCTC
MS4A15	F	AATCCCGCCTTTGTTTCCCA
	R	CTGGAGGTTGGCACATGGAT
GSTA1	F	TGATCCTCCTTCTGCCCGTA
	R	CAAAGGCAGGGAAGTAGCGA
KRT6A	F	AGCACTAAAGTGCGTCTGCT
	R	GTGAGCAATGGGTGCTCAGA
Actin	F	GTGGATCAGCAAGCAGGAGT
	R	ATCCTGAGTCAAGCGCCAAA
